# Supplementary material for: Cell wall composition and lignin biosynthetic gene expression along a developmental gradient in an Australian sugarcane cultivar
Source: PeerJ. 2017 Dec 5;5:e4141. doi: 10.7717/peerj.4141 (PMC5721908; doi:10.7717/peerj.4141)
Supplement: Table S1 — qRT-PCR analysis was performed on lignin biosynthesis gene expression levels in five stem sections (A–E) and roots (n = 5 per tissue section per gene). Results represent raw data before normalization. [file peerj-05-4141-s001.docx]

| **Gene** | **section A** | | **section B** | | **section C** | | **section D** | | **section E** | | **Roots** | |
| --- | --- | --- | --- | --- | --- | --- | --- | --- | --- | --- | --- | --- |
|  | ΔCt | +/- | ΔCt | +/- | ΔCt | +/- | ΔCt | +/- | ΔCt | +/- | ΔCt | +/- |
| **PAL** | 97.57 | 15.63 | 21.11 | 5.81 | 8.00 | 1.73 | 6.05 | 1.56 | 3.46 | 1.01 | 97.56 | 7.50 |
| **C4H** | 0.21 | 0.03 | 0.26 | 0.02 | 0.22 | 0.01 | 0.24 | 0.02 | 0.28 | 0.02 | 1.90 | 0.12 |
| **C3H** | 0.08 | 0.01 | 0.19 | 0.04 | 0.14 | 0.02 | 0.11 | 0.01 | 0.13 | 0.02 | 0.14 | 0.01 |
| **4CL** | 0.31 | 0.09 | 0.11 | 0.01 | 0.09 | 0.01 | 0.12 | 0.02 | 0.11 | 0.01 | 0.53 | 0.06 |
| **CCoAOMT** | 33.79 | 6.08 | 21.89 | 3.83 | 27.45 | 4.32 | 24.81 | 4.38 | 28.50 | 4.07 | 24.30 | 1.17 |
| **F5H** | 0.17 | 0.05 | 0.32 | 0.04 | 0.20 | 0.05 | 0.23 | 0.08 | 0.22 | 0.05 | 0.21 | 0.03 |
| **CAD** | 1.66 | 0.38 | 0.97 | 0.16 | 0.42 | 0.10 | 0.45 | 0.13 | 0.64 | 0.12 | 1.06 | 0.16 |
| **CCR** | 14.68 | 3.37 | 4.11 | 1.02 | 2.41 | 0.55 | 2.19 | 0.73 | 1.30 | 0.47 | 11.50 | 0.92 |
| **COMT** | 24.79 | 4.78 | 9.08 | 1.85 | 6.16 | 0.95 | 5.96 | 2.09 | 6.59 | 0.82 | 15.31 | 0.96 |
