# Supplementary figures and images for: Cell wall composition and lignin biosynthetic gene expression along a developmental gradient in an Australian sugarcane cultivar
Source: PeerJ. 2017 Dec 5;5:e4141. doi: 10.7717/peerj.4141 (PMC5721908; doi:10.7717/peerj.4141)

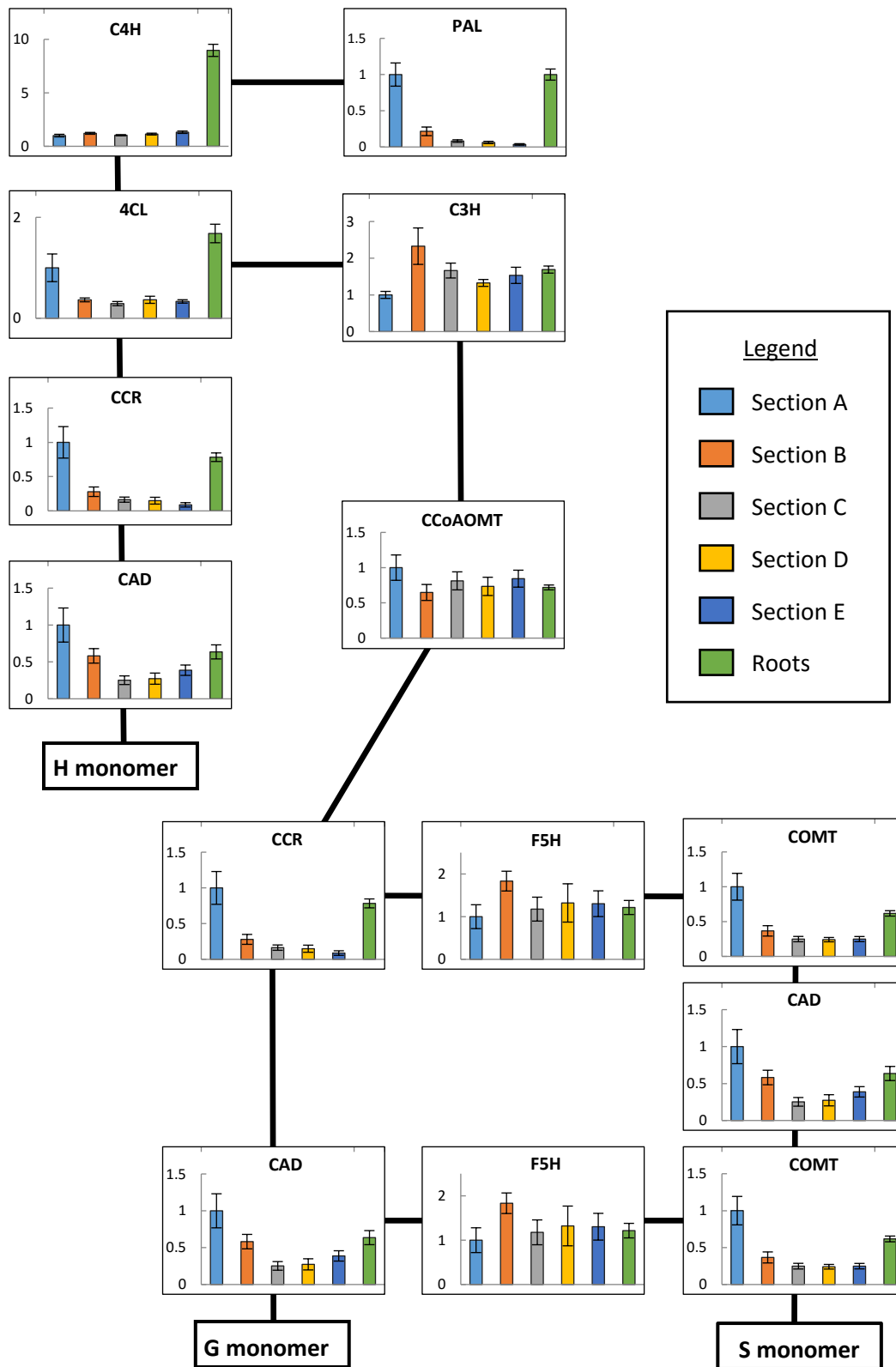

Supplement: Figure S1 — Gene expression in stem sections A–E and root tissue as seen in Figs. 1 and 2 laid out as lignin biosynthesis pathway. Expression levels are normalized to section A for each gene. [file peerj-05-4141-s002.pdf]
